# Editorial: *Aspergillus*-Derived Mycotoxins in the Feed and Food Chain

**DOI:** 10.3389/fmicb.2020.606108

**Published:** 2020-11-11

**Authors:** István Pócsi, Federica Giacometti, Árpád Ambrus, Antonio F. Logrieco

**Affiliations:** ^1^Department of Molecular Biotechnology and Microbiology, Faculty of Science and Technology, Institute of Biotechnology, University of Debrecen, Debrecen, Hungary; ^2^Department of Veterinary Medical Sciences, University of Bologna, Bologna, Italy; ^3^Doctoral School of Nutrition and Food Sciences, University of Debrecen, Debrecen, Hungary; ^4^Institute of Sciences of Food Production, Italian National Research Council, Bari, Italy

**Keywords:** aflatoxins, ochratoxin A, *Aspergilli*, food chain, mycotoxicoses, risk assessment, climate change

*Aspergillus*-produced mycotoxins can enter the feed and food chain at many points in both pre-harvest and post-harvest. Although current climate changes seem to speed up the world-wide spread of mycotoxigenic fungi including the *Aspergilli* and also facilitate the production of these harmful secondary metabolites the factors governing these disadvantageous global processes are only partly understood or even have remained completely hidden until now. This Research Topic summarizes our knowledge on *Aspergillus*-derived mycotoxins especially focusing on *three* major areas of on-going research: (i) toxicological, medical, veterinary aspects, prevalence, detection, risk assessment, control strategies, (ii) ecology and biological control of mycotoxigenic *Aspergilli* in the fields, and (iii) pre-harvest and post-harvest management of mycotoxigenic *Aspergilli* and their mycotoxin production. We hope that the wealth of information generously provided by the *Aspergillus* mycotoxin research community will help the hard work of all those experts, who are active in this important field, and the papers collected here will be instructive and illuminating readings for students and the public as well.

## Toxicological, Medical, Veterinary Aspects, Prevalence, Detection, Risk Assessment, Control Strategies

### Toxicological, Medical and Veterinary Aspects

*Aspergillus*-derived mycotoxins (aflatoxins, ochratoxins, gliotoxin, fumonisins, sterigmatocystin, patulin, etc.) represent a remarkably versatile group of fungal secondary metabolites considering both their chemical structures and adverse physiological effects in humans (Ráduly et al.). Although current food safety measures are often adequate to prevent the accumulation of these mycotoxins in the food chain further interdisciplinary research is eagerly needed to elaborate more effective prevention strategies of mycotoxicoses, to reach a deeper understanding of the deleterious consequences of both sole and combined mycotoxin exposures at various stages of life, and to invent novel diagnostic tools and therapeutic procedures to mitigate both acute and chronic mycotoxin poisonings (Ráduly et al.). To prevent primary and secondary (*via* food of animal origin) aflatoxicoses in humans is of paramount importance (Peles et al.). Fortunately, the accurate physiological effects, the existent transformation and detoxification pathways and the mechanisms of channeling of harmful aflatoxins into food raw materials have been elucidated in important livestock like poultry, pigs and ruminants, and a wide spectrum of biocontrol and detoxification products are available to prevent harmful aflatoxins from entering the feed and food chain in animal husbandry (Peles et al.).

### Prevalence and Detection of Aflatoxins

Food and feed contamination by aflatoxins create food insecurity around the world. Two overviews detail the prevalence of *Aspergillus* section *Flavi* and the occurrence of aflatoxins in raw peanuts and peanut-based products (Norlia et al.) and in food and feed (Mahato et al.). Since even a low aflatoxin concentration is hazardous for human and livestock, the identification and quantification of AFs is a major challenge to guarantee food safety. The demand for determination of aflatoxins triggered extensive research and method development, and, in the last decades, increasingly faster and more sensitive analytical techniques proved to be promising, but, only a few of them have gained applicability in routine analysis. The study of Miklós et al. provides guidance on the current performance characteristics of various analytical methods for determination of aflatoxins in different food and feed matrices, and highlights their limitations for practical use, i.e., the absence of processes applied for reduction of large laboratory samples to the few grams for extraction (Miklós et al.) or the fact that the repeatability or reproducibility, if reported, was based on a few spiked samples (Miklós et al.). This guide helps in the decision to choose the most appropriate method that meets the practical requirements of fast and sensitive control of their contamination. Special references are devoted to new methods developed for masked AFs that are unable to be identified by routine analysis processes (Mahato et al.) and for concomitant detection of aflatoxins and their major fungi precursors *Aspergillus flavus* and *Aspergillus parasiticus* in stored maize by a simultaneous run of a Display Mediated Immuno-polymerase Chain Reaction (PD-IPCR) for aflatoxins and a conventional real-time PCR (RT-PCR) for aflatoxin producers (Ren, Yue et al.).

### Risk Assessment and Control Strategies for Aflatoxins

Despite of prevention methods and strict regulations, *Aspergillus-*derived mycotoxins are still present in the feed and food chain, as well as the mycotoxicoses (Ráduly et al.). Quantitative exposure assessment is a methodology developed to evaluate the probable intake of chemical substances *via* food. The study of Serraino et al. calculated the Estimated Daily Intake, the Hazard Index, and the fraction of hepatocarcinoma cases (HCC) due to AFM_1_ exposure in different population groups in Italy. A low risk of HCC was predicted but the variability of climatic conditions throughout years justifies a continuous monitoring of aflatoxins and an update of the risk assessment. To implement appropriate control measures, a special focus is devoted to the aflatoxin management and the impact of climate change on AFs production, and of control strategies of AFs in terms of innovative processing technologies applied for pre-and post-harvest aflatoxins management in combination with either biological, physical, chemical or genetic engineering methods (Mahato et al.; Ráduly et al.).

## Ecology and Biological Control of Mycotoxigenic *Aspergilli* In The Fields

### Interactions of the *Aspergilli* and Their Mycotoxins With Plants and the Soil Micro-and Macrobiota

The remarkably complex and dynamic network of soil microbiota and macrobiota determining the ecological niches the mycotoxigenic *Aspergilli* can enter and fill in is still waiting to be described and deciphered in details (Pfliegler et al.). Ecological factors influencing the production and fate of fungal secondary metabolites including mycotoxins like sterigmatocystin/aflatoxins, gliotoxin, ochratoxins, patulin, and cyclopiazonic acid as well as the versatile interactions of these molds with plants (e.g., *A. flavus* with peanut, maize, and cotton), other microorganisms (including fungi, prokaryotes, and protists) and animals (first of all with arthropods) need to be clarified to reach a deeper understanding of these ecosystems and also to develop novel biocontrol and mycotoxin biodegradation technologies for plant and food protection (Pfliegler et al.). In the last decades, various and powerful omics techniques helped us to shed light on the fine details of the molecular mechanisms of host-pathogen interactions especially in *A. flavus*-maize and *A. flavus*-groundnut relations (Soni et al.). Not surprisingly, a plethora of proteins and genes have been identified with definite or hypothesized functions in the resistance of these agricultural crops to aflatoxin contaminations, which has opened the way to the development of novel molecular breeding technologies in this important field (Soni et al.).

### Monitoring Atoxigenic Biocontrol *Aspergillus flavus* Genotypes in Fields

Application of non-aflatoxigenic *A. flavus* strains to prevent aflatoxin contamination under field conditions is one of the leading pre-harvest strategies to control this harmful, carcinogenic mycotoxin in the feed and food chain. Interactions of these biocontrol agents with the indigenous soil populations of aflatoxigenic fungi has been the subject of extensive research for a long time. Interestingly, the Afla-Guard strain originally isolated from naturally infected peanut in Georgia and belonging to lineage IB performed better in maize fields in the south-eastern United Stated (Alabama, Georgia, North Carolina) than the AF36 strain in lineage IC and isolated from cottonseed in Arizona as indicated by shifts in genetic diversities (Lewis et al.). In Ghana, 12 atoxigenic African *A. flavus* VCGs (AAVs) were identified and the biocontrol potential of a representative member of each AAV was tested under both laboratory (maize) and field (maize and groundnut, in three diverse agroecological zones) conditions (Agbetiameh et al.). As a result, four-four well-preforming isolates were selected and incorporated into two biocontrol products, Aflasafe GH01 and Aflasafe GH02, for use in maize and groundnut cropping systems in Ghana (Agbetiameh et al.). Importantly, each isolate has a unique simple sequence repeat (SSR) signature based on 17 SSR loci, which makes the tracking of each active ingredient possible under field conditions (Agbetiameh et al.). Fast and reliable tracking and quantification of active ingredients of biocontrol products in crops are also required by farmers, the regulatory community and crop end-users (Shenge et al.). An array of quantitative pyrosequencing-based assays was developed and successfully applied in maize-associated fungal populations to monitor frequencies of SNPs characteristic of non-aflatoxigenic *A. flavus* isolates included in the Aflasafe product in Nigeria (Shenge et al.).

### Biopesticides and Biostimulants

The use of biological agents and biostimulants for the control of *A. flavus* is a prerequisite for creating an Integrated Pest Management (IPM) in order to protect plants and related products prone to aflatoxins contamination. Commercial biopesticides could offer an economically effective solution that may contribute to the exclusion of aflatoxigenic fungi from maize plants and minimize the mycotoxin production. The efficiency evaluation of these biopesticides *in vitro* assay is crucial for a future IPM system friendly and sustainable for the environment (Lagogianni and Tsitsigiannis; Lagogianni and Tsitsigiannis).

## Pre-Harvest and Post-Harvest Management of Mycotoxigenic *ASPERGILLI* and Their Mycotoxin Production

### Regulation of Mycotoxin Biosynthetic Gene Clusters

The remarkable complexity of aflatoxin biosynthesis has been revealed in aflatoxigenic fungi and both biotic and abiotic factors contribute to the fine-tuning of mycotoxin production in these microorganisms (Peles et al.; Pfliegler et al.). For example, abiotic oxidative stress stimulates aflatoxin biosynthesis in *A. flavus* (Peles et al.), and 3.5% ethanol exposure of *A. flavus* colonies not only significantly down-regulated the great majority of the aflatoxin cluster genes but also up-regulated important elements of the oxidative stress defense system including the *Cat, Cat1, Cat2, CatA*, and *sod1* genes as well as the oxidative stress response regulator genes *ap-1* and *msnA* (Ren, Jin et al.). These transcriptional changes coincided with a nearly complete inhibition of aflatoxin B_1_ production (Ren, Jin et al.).

The ceaseless demand for the development of novel mycotoxin control technologies also requires further research to be performed on the regulation of mycotoxin biosynthetic gene clusters (Peles et al.). Wang et al. reported on the pivotal role the velvet complex (consisting of LaeA, VeA, and VelB proteins) of *Aspergillus ochraceus* plays in the maintenance of vegetative growth, asexual sporulation, virulence (on pears), and ochratoxin A production of the fungus and, therefore, elements of the velvet complex seem to be attractive targets for future ochratoxin A control technologies. It is worth noting that LaeA extensively regulates the secondary metabolism of *A. ochraceus* (Wang et al.). Another study by Barda et al. shed light on the remarkable importance of the pH-responsive transcription factor AcPacC on the regulation of fungal growth at neutral/alkaline pH, asexual sporulation, spore germination, gluconic and citric acid productions, ochratoxin A production (also on grapes and nectarine fruits) and virulence of *Aspergillus carbonarius*. Importantly, glucose oxidase encoded by *Acgox* was demonstrated to be a virulence factor of *A. carbonarius* and its production was also under a strict AcPacC control (Barda et al.).

### Plant Bioactive Compounds Against Mycotoxigenic Fungi

Bioactive metabolites of plants like phenolic compounds, terpenes and nitrogen-containing compounds may also possess antifungal activities *via* interfering cell wall and cell membrane biosynthesis, mitochondrial functions, and important enzyme activities in fungi (Loi et al.). In addition to controlling pre-harvest and post-harvest growths of the *Aspergilli*, the direct inhibitory effects of plant bioactive compounds on aflatoxin biosynthetic pathway seem to be exploitable as well (Loi et al.). Various plant extracts containing low molecular weight active ingredients and enzymes have also been shown to be effective in aflatoxin degradation (Loi et al.). Cinnamaldehyde, a widely used α,ß-unsaturated aldehyde food additive, appears to be especially promising in control of aflatoxigenic *A. flavus* e.g., on corn (Qu et al.). Considering the antifungal mechanism of action of this compound, cinnamaldehyde triggers a series of apoptotic events in *A. flavus*, including elevated intracellular Ca^2+^ and reactive oxygen species levels, various mitochondrial dysfunctions, metacaspase activation, phosphatidylserine externalization, DNA damage and nuclear fragmentation as well as up-regulation of apoptosis-related genes (Qu et al.). A methanolic pomegranate peel extract (PPE) acted synergistically with the azole fungicide prochloraz (PRZ) in controlling the growths of the mycotoxigenic fungi *A. flavus* and *Fusarium proliferatum* (Sadhasivam et al.). PPE+PRZ combined treatments delayed conidial germination and hyphal elongation in both fungi, and the combined application of sub-inhibitory doses of PPE and PRZ blocked aflatoxin B1 production by *A. flavus* (Sadhasivam et al.). Such combined antifungal treatments may help us to decrease the applied doses of potentially harmful synthetic fungicides like the azole drugs in the agriculture (Sadhasivam et al.).

### Fungal Biomass as Aflatoxin Biosorbent

Adsorbent materials mixed with the feed may protect animals by binding efficiently mycotoxins including aflatoxins. The adsorbents reduce the bioavailability of mycotoxins in the gastro-intestinal tract and thus the diffusion into the bloodstream and transport to the target organs. The use of microbial biomasses as adsorbent seems very promising since less expensive though effective, and environmentally friendly materials (Haidukowski et al.). The characterization of the bio-sorbent properties of the mycelium of the king oyster mushroom (*Pleurotus eriyngii*) as for Aflatoxin B_1_ binding capability and the effects of physical and chemical conditions on the binding efficiency is strategic for a sustainable mycotoxin risk minimization in animal health (Haidukowski et al.).

## Author Contributions

All authors served as co-editors to the Research Topic and also contributed to, critically read and discussed and approved this Editorial.

## Conflict of Interest

The authors declare that the research was conducted in the absence of any commercial or financial relationships that could be construed as a potential conflict of interest.

